# Inhibition of Tumor Growth and Immunomodulatory Effects of Flavonoids and Scutebarbatines of *Scutellaria barbata* D. Don in Lewis-Bearing C57BL/6 Mice

**DOI:** 10.1155/2015/630760

**Published:** 2015-05-03

**Authors:** Tao Gong, Chun-Fei Wang, Jia-Rui Yuan, Yu Li, Jun-Fei Gu, Bing-Jie Zhao, Li Zhang, Xiao-Bin Jia, Liang Feng, Shen-Lin Liu

**Affiliations:** ^1^Department of Oncology, The Affiliated Hospital of Nanjing University of Chinese Medicine, No. 155, Hanzhong Road, Nanjing 210029, China; ^2^Key Laboratory of New Drug Delivery Systems of Chinese Materia Medica, Jiangsu Provincial Academy of Chinese Medicine, Shi Zi Street No. 100, Hongshan Road, Jiangsu, Nanjing 210028, China; ^3^Anhui University of Chinese Medicine, Hefei 230038, China; ^4^School of Pharmacy, Jiangsu University, Zhenjiang 212013, China

## Abstract

Immunomodulatory effect has been found to be an important therapeutic measure for immune responses against cancer. In this study, we evaluated the inhibition of* Scutellaria barbata* D. Don (SB), an anti-inflammatory and an antitumor Chinese herb, including flavonoids and scutebarbatines on tumor growth and its immunomodulatory effects* in vivo*. HPLC and LC/MS/MS methods were conducted for the analysis of flavonoids and scutebarbatines in SB. Lewis-bearing C57BL/6 mice model was established and tumor volume was evaluated by high frequency color ultrasound experiment. ELISA and western blot analysis were performed for the determination of immunomodulatory factors. SB treatment at the dose of 10, 6.67, and 3.33 g crude drug/kg/d significantly inhibited tumor growth of Lewis-bearing C57BL/6 mice with the inhibition rates of 44.41 ± 5.44%, 33.56 ± 4.85%, and 27.57 ± 4.96%, respectively. More importantly, the spleen and thymus indexes were increased remarkably by SB treatment. SB could decrease IL-17, IL-10, FOXP3, TGF-*β*1, ROR*γ*t, and IL-6 levels whereas it could increase remarkably IL-2 and IFN-*γ* levels. Our results demonstrated that SB could inhibit tumor growth* in vivo* through regulating immune function in tumor-bearing mice and suggested that the immunomodulatory function of SB had a potential therapeutic effect in lung cancer.

## 1. Introduction

The high morbidity and mortality of lung cancer which represent the first leading cause of death in the world have become an important health challenge of public concern [1]. Besides the traditional therapy measures, such as radiotherapy and chemotherapy, the immunotherapy is becoming the focus of interest of researchers [2]. The recent study provided evidence for the possible application of immunotherapy for the treatment of non-small-cell lung cancer (NSCLC) in early and advanced stages [3]. There is a growing body of evidence indicating that the regulation of T cells can prolong the survival of patients with NSCLC [4, 5]. Recent developments of immunotherapy may provide clinical benefit in the treatment of lung cancer.

Traditional Chinese medicine (TCM) is helpful for cancer treatment due to its immunomodulatory function.* Scutellaria barbata* D. Don (SB), belonging to* Labiatae*, is a traditional Chinese herb and is widely distributed in Korea and southern China. Its dry rhizomes have been used as an anti-inflammatory and antitumor medicinal herb for the use of treatment of tumors or cancers in China. Extracts of SB have been shown to hold inhibitory effects* in vivo *on a variety of tumors [6]. SB is commonly used to treat breast cancer [7], colorectal cancer [8], hepatocarcinoma [9], lung cancer [10], and so forth.

In recent years, some researches have demonstrated that flavonoids and scutebarbatines are the main components of SB [11, 12]. The research showed that the flavonoids of SB had significant antitumor activity due to the enhancement of the immune function [13]. Flavonoids of SB including scutellarin, naringin, apigenin, luteolin, and wogonin have been found to significantly inhibit the proliferation and invasion of MHCC97H cells in a dose-dependent manner [14]. Yang et al. demonstrated that scutebarbatine A possessed significant antitumor effects on A549 cells* in vitro* and transplanted tumor nude mice* in vivo* via mitochondria-mediated apoptosis [15]. However, there are few researches on the immunomodulatory function of SB on the tumor growth of lung cancer.

Therefore, the aim of this study was to evaluate the inhibition of tumor growth and immunomodulatory effects of SB including flavonoids and scutebarbatines in Lewis-bearing C57BL/6 mice. Our data indicated that SB could inhibit tumor growth of Lewis-bearing C57BL/6 mice through modulating the immune function. Our findings provided experimental evidence for the application in the treatment of lung cancer.

## 2. Materials and Methods

### 2.1. Materials

Fetal bovine serum (FBS), RPMI 1640 medium, and trypsin were purchased from Gibco/BRL (Grand Island, NY, USA). Dimethyl sulfoxide (DMSO) was purchased from Sigma Chemical (St. Louis, MO, USA). Cisplatin (DDP), streptomycin, and penicillin were purchased from Nanjing Pharmaceutical Co., Ltd. (Nanjing, China). HPLC grade methanol, acetonitrile, and phosphoric acid used as mobile phase were obtained from TEDIA (Fairfield, OH, USA). ELISA kits for IL-10, IL-17, FOXP3, IFN-*γ*, IL-2, ROR*γ*t, TGF-*β*1, and IL-6 were purchased from KeyGEN Biotech. Co., Ltd. (Nanjing, China). Antibodies to these immunoregulatory factors including IL-10, IL-17, FOXP3, IFN-*γ*, IL-2, ROR*γ*t, TGF-*β*1, IL-6, and *β*-actin were obtained from Wuhan BOSTER Biological Engineering Co., Ltd. (Wuhan, China). The standard substances including naringin, scutellarin, scutebarbatine B, scutebarbatine A, wogonin, luteolin, scutellarein, and apigenin (the content ≥ 98%) were ordered from National Institutes for Food and Drug Control (Beijing, China). Remaining reagents were of analytical grade.

### 2.2. Preparation of SB Extract

The dried rhizomes of SB were purchased from Nanjing Haiyuan Chinese medicine decoction pieces Co., Ltd. (Nanjing, China). And they were authenticated by Professor Q. N. Wu, from the Nanjing University of Chinese Medicine. The specimens were stored in our laboratory under standard conditions. The dried rhizomes of SB were grounded into powder. The powder (1 kg) was weighed and extracted with double-distilled water (1000 mL) by reflux extraction for 1.5 h/time 2 times. The combined extract was added with 95% ethanol (v/v) to adjust the final concentration of ethanol to 85% (v/v). The precipitated polysaccharide component was removed by the filtration device. The remaining solution was concentrated at 50°C in a rotary evaporator under reduced pressure. The yield of final extract was 1.14% while the purity was 81.07%. Finally, the extract was redissolved in methanol for HPLC-DAD and LC-ESI-MS/MS analysis. The supernate of the samples was filtered through 0.45 *μ*m cellulose acetate membrane.

### 2.3. HPLC and LC/MS/MS Analysis

The extract of SB was analyzed by Agilent 1200 series HPLC instrument (Agilent, Wilmington, DE, USA). The sample was separated on an Agilent Eclipse TC-C18 column (4.6 mm × 150 mm, 5 *μ*m). The mobile phase gradient conditions consisted of acetonitrile (A) and water (B): 0–2 min, 10%-10%A; 2–20 min, 10%-18%A; 20–40 min, 18%-18%A; 40–60 min, 18%-25%A; 60–70 min, 25%-50%A; 70–110 min, 50%-95%A; 110–120 min, 95%-95%A. The flow rate was 0.8 mL/min and the column temperature was maintained at 25°C. Sample of 10.0 *μ*L was detected at 264 nm.

LC/MS/MS analysis was performed on Thermo Fisher Scientific ion trap mass spectrometer, equipped with an electrospray ionization (ESI) interface (Bremen, Germany). This analysis was operated under negative-ion mode. The optimized operating parameters were as follows: ion spray voltage, 4 kV; the nebulizer gas (GS1): 0.45 L/min; curtain gas (CUR): 0.2 L/min; declustering potential 1 (DP1): 20 V; focusing potential (FP): 80 V; declustering potential 2 (DP2): 20 V. The mass spectrometer was detected over a range of *m*/*z* 80 to 1500 in the full scan mode.

### 2.4. Cell Culture

Lewis mice cell line was purchased from Chinese Academy of Medical Sciences of tumor institute (Shanghai, China). Lewis cells were incubated in DMEM medium, supplemented with 10% fetal bovine serum (FBS), 100 U/mL of penicillin, and 100 *μ*g/mL of streptomycin. Cells were maintained in a humidified incubator at 5% CO_2_ and 37°C. The medium was renewed every 2 days. These cells were detached by 0.25% trypsin-0.01% EDTA and used for seeding. Cells which were passaged for 3 to 4 times were used for experiment.

### 2.5. Lewis Tumor-Bearing C57BL/6 Mice and Treatment

Male C57BL/6 mice (18–22 g) were purchased from SLAC experimental animals Co., Ltd. (Shanghai, China). The animal experiment protocol was reviewed and approved by the Institutional Animal Care and Use Committee of the Jiangsu Provincial Academy of Chinese Medicine. Lewis cells (1 × 10^6^ cells/mL) were injected subcutaneously in right armpit with 0.2 mL. Then the mice were randomly divided into six groups and administered for 2 consecutive weeks: blank control; model group; positive control DDP (20 mg/kg/d) group; SB (10, 6.67, and 3.33 g crude drug/kg/d). Blank and model mice were treated orally with saline solution (0.9%) each day. Two weeks later, the blood samples which were taken from retinal vein were determined with ELISA kits. After being sacrificed, the tumors were weighed immediately. The inhibition ratio was calculated using the following formula: inhibitory ratio (%) = [(the average tumor weights of the model − the average tumor weights of treated groups)/the average tumor weights of the model] × 100% [16]. The spleen index and thymus index were calculated using the following formula: spleen index = the weights of spleen/the weights of C57BL/6 mice; thymus index = the weights of thymus/the weights of C57BL/6 mice.

### 2.6. Animal Dedicated High Frequency Color Ultrasound

The tumor volume was detected by animal dedicated high frequency color ultrasound on 14th day. 3D tumor volumes were determined by VisualSonics Vevo2100 Animal dedicated high frequency color ultrasound system (Toronto, ON, Canada). Instrument parameter was set as follows: the probe was the MS-400, and its frequency was 30 Hz. The detection was kept at the probing depth of 8 mm and a width of 10.36 mm. After being anesthetized with inhalation of 2% isoflurane, mice were fixed on a hot plate at 37°C. The probe was placed to the tumor at the right armpit of mice. The area of each section was recorded and then three sections were combined into a three-dimensional map to calculate tumor volume.

### 2.7. ELISA for Immunomodulatory Factors

The potential immunomodulatory activities of SB including flavonoids and scutebarbatines were determined by ELISA. The blood samples were collected from retinal vein and centrifuged at 4000 ×g at 4°C for 15 min; the supernatant was taken for the determination with ELISA. The detailed procedures were performed according to the manufacturers' instructions. After the reaction was stopped, the absorbance was measured with a microplate reader (SPECTRAmax19.0, Molecular Devices, USA). The detection wavelength was fixed at 450 nm with a reference wavelength at 655 nm. The levels of immunomodulatory activities were calculated according to their standard curve.

### 2.8. Western Blot Assay

Tumor tissue was taken and then washed twice with PBS (pH = 7.2). Sequentially, tissues were lysed for 30 min at 4°C with ice-cold RIPA buffer containing 1% Nonidet P-40, 50 mM Tris, and 2 mM ethylenediaminetetraacetic acid [EDTA], 120 mM NaCl, 11.5 *μ*g/mL aprotinin, 11.5 *μ*g/mL leupeptin, 50 *μ*g/mL phenylmethylsulfonyl fluoride, 100 mM sodium fluoride, and 0.2 mM sodium orthovanadate. The concentration of protein was quantified by the bicinchoninic acid (BCA) method according to the manufacturer's instructions (Pierce, Rockford, USA). Then, the equalized amounts of proteins from each sample were resolved in sodium dodecyl polyacrylamide gel electrophoresis (SDS-PAGE) and transferred to Polyvinylidene Fluoride (PVDF). After being blocked with 5% bovine serum albumin (BSA) for 2 h, washed in TBST thrice, these membranes were incubated with primary antibodies against IL-10, IL-17, FOXP3, IFN-*γ*, IL-2, ROR*γ*t, TGF-*β*1, and IL-6 (1 : 500) overnight at 4°C and then incubated with the secondary antibody conjugated with horseradish peroxidase for 1 h at room temperature. Finally, the protein bands were treated with enhanced chemiluminescent reagents for the visualization. *β*-actin (1 : 500) was used as the internal reference for loading control.

### 2.9. Statistical Analysis

All data from the different groups were analyzed using SPSS 11.5 software (IBM, Armonk, NY, USA). One-way analysis of variance was used for multiple comparisons, and Student's *t*-test was used to compare two groups. A value of *p* < 0.05 was considered statistically significant. All values were expressed as means ± standard deviation (SD).

## 3. Results 

### 3.1. Identification of Chemical Components

**Figure 1:**
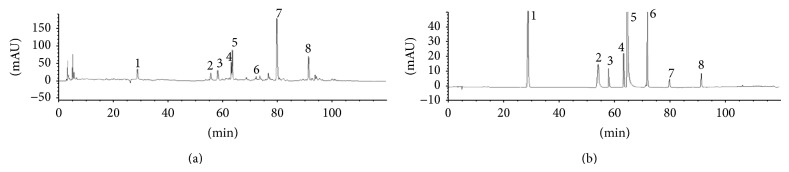
The HPLC chromatogram of flavonoids and scutebarbatines of SB. (a) SB extract; (b) mixed standard substances. 1: scutellarin; 2: naringin; 3: scutellarein; 4: luteolin; 5: apigenin; 6: wogonin; 7: scutebarbatine A; 8: scutebarbatine B.

After being dissolved with methanol, the main compounds were analyzed and identified by high performance liquid chromatography-diode array detection (HPLC-DAD) and liquid chromatography-electrospray ionization-tandem mass spectrometry (LC-ESI-MS/MS). The HPLC-DAD chromatogram of SB was shown in Figure 1. The chromatogram showed that flavonoids and scutebarbatines of SB could be eluted completely under the used HPLC conditions within 120 min and separated satisfactorily. The peaks whose retention time ranges from 20 min to 100 min might consist mainly of flavonoids and scutebarbatines, compared with these standard substances.

**Figure 2:**
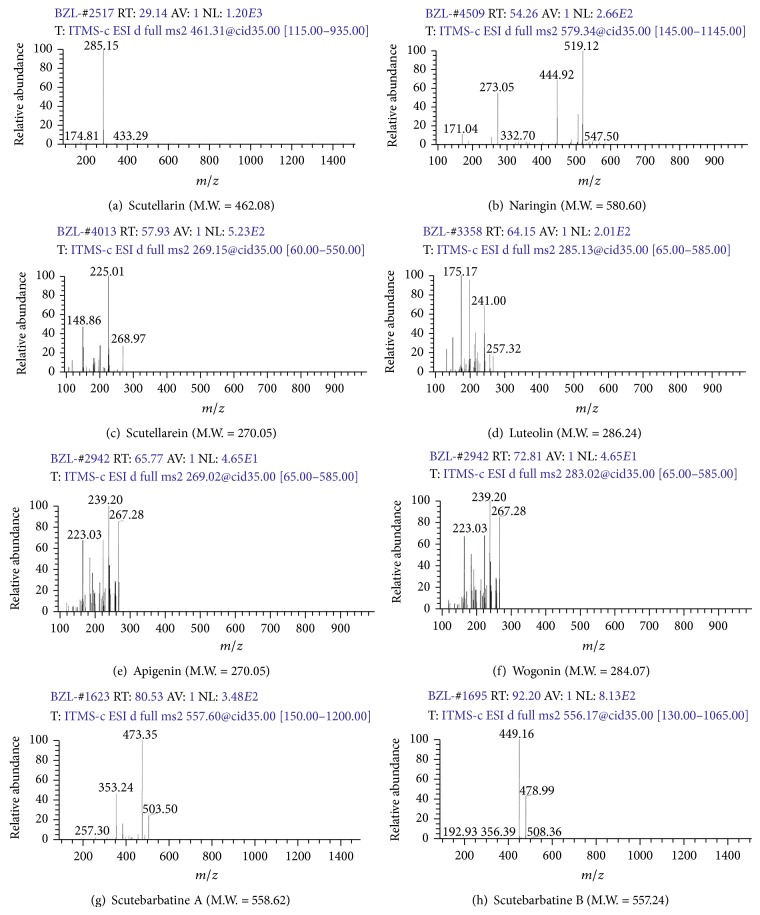
LC-ESI-MS/MS spectra for flavonoids and scutebarbatines of SB.

**Figure 3:**
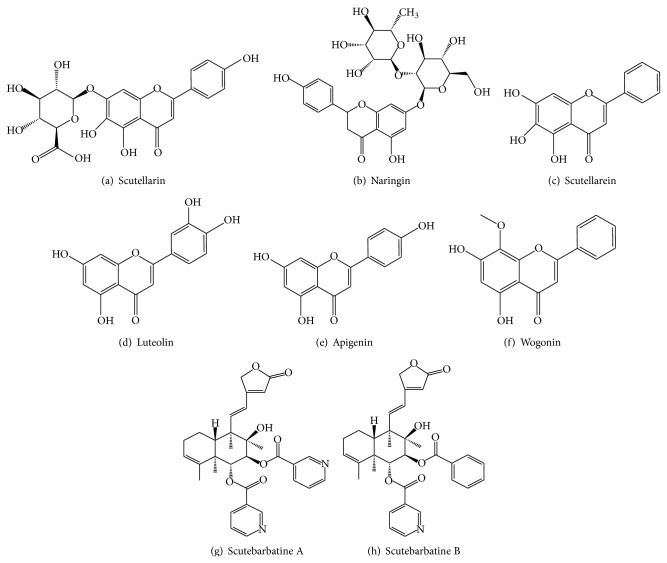
The chemical structure of flavonoids and scutebarbatines of* Scutellaria barbata *D. Don.

In order to verify these compounds, HPLC-ESI-MS/MS method was conducted to identify these compounds according to fragment ion peaks. As shown in Figure 2, these compounds of SB were identified as scutellarin; naringin; scutellarein; luteolin; apigenin; wogonin; scutebarbatine A; scutebarbatine B (Figure 3 and Table 1). Our established HPLC-DAD and HPLC-ESI-MS/MS methods could simultaneously identify the multiple bioactive components of flavonoids and scutebarbatines in SB.

### 3.2. Effects of Flavonoids and Scutebarbatines in SB on Tumor Growth, Tumor Volume, and Tumor Weight in C57BL/6 Mice

**Figure 4:**
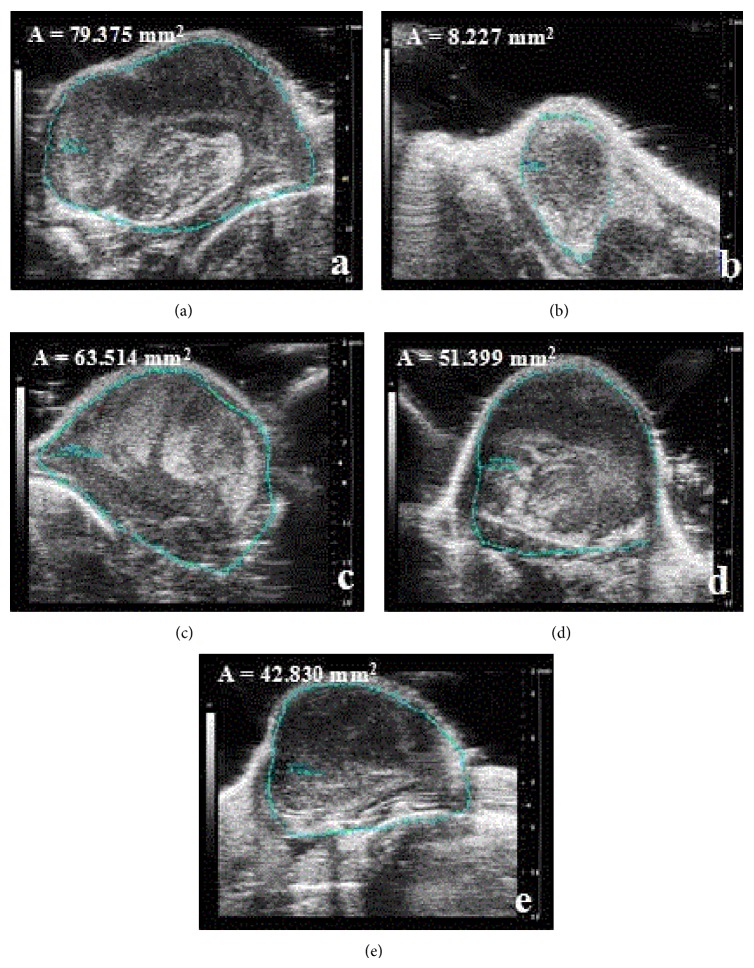
Animal dedicated high frequency color ultrasound pictures for tumor growth in Lewis-bearing C57BL/6 mice. Mice were treated with SB (3.33, 6.67, and 10 g crude drug/kg/d) including flavonoids and scutebarbatines for continuous 7 days. (a) Model; (b) positive control DDP (20 mg/kg/d); ((c)–(e)) SB (3.33, 6.67, and 10 g crude drug/kg). The area of tumor (A) was calculated. Data were presented as means ± SD (*n* = 10).

**Figure 5:**
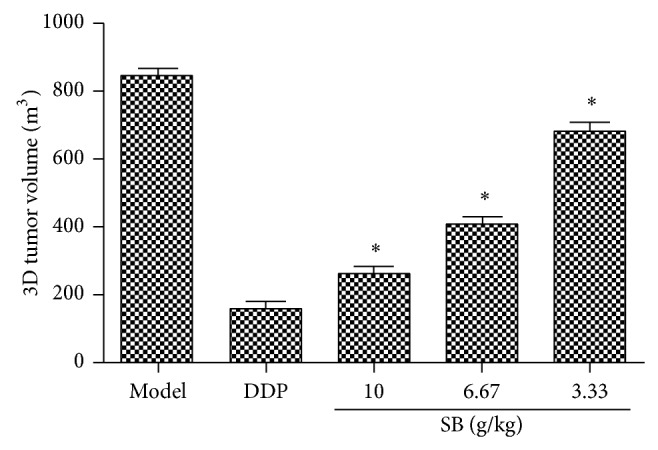
3D tumor volume of tumor-bearing C57BL/6 mice by animal dedicated high frequency color ultrasound. All data were expressed as means ± SD (*n* = 10). ^∗^ *p* < 0.05, versus model group.

Lewis tumor-bearing C57BL/6 mice were used to verify the anticancer activities of SB including flavonoids and scutebarbatines. The treatments of SB (3.33, 6.67, and 10 g crude drug/kg/d) were started on the 2nd day after these mice were injected with tumor cells. After being administered orally for continuous 7 days, the tumor blocks in right anterior limb could be touched. Then, tumor volumes were determined by animal dedicated high frequency color ultrasound system. As the results shown in Figures 4 and 5, the area and 3D tumor volumes of SB-treated mice were lower than those of model mice in a dose-dependent manner (*p* < 0.05). Furthermore, the positive control Cisplatin (DDP) at the concentration of 20 mg/kg/d could reduce significantly the 3D tumor volumes, compared with the model mice (*p* < 0.05).

**Figure 6:**
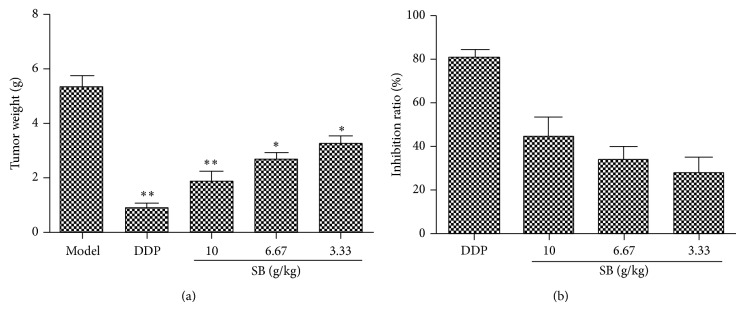
Effect of SB on tumor weight and inhibition rate in Lewis tumor-bearing C57BL/6 mice. (a) Tumor weight; (b) inhibition rate of tumor growth. All data were expressed as means ± SD (*n* = 10). ^∗^ *p* < 0.05, ^∗∗^ *p* < 0.01, versus model group.

As shown in Figure 6(a), a significant decrease of tumor weight in SB (3.33, 6.67, and 10 g crude drug/kg/d) treated group was observed compared to the model mice (*p* < 0.05). SB treatment significantly inhibited tumor weights in mice by 44.41 ± 5.44%, 33.56 ± 4.85%, and 27.57 ± 4.96%, compared with the model group (*p* < 0.05) (Figure 6(b)). Our findings suggested that SB had a significant tumor inhibition effect on tumor-bearing mice.

### 3.3. Immunomodulatory Effects of Flavonoids and Scutebarbatines in SB on Thymus Index and Spleen Index of Tumor-Bearing C57BL/6 Mice

**Figure 7:**
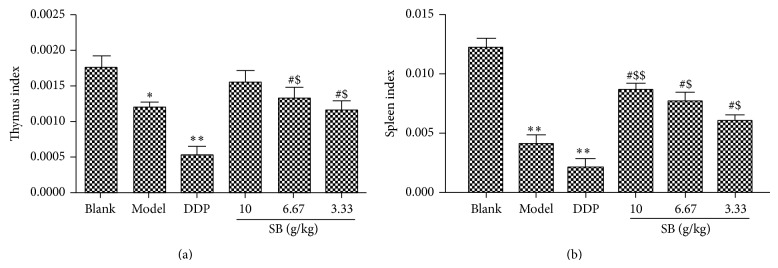
Effect of flavonoids and scutebarbatines on spleen index and thymus index in Lewis tumor-bearing C57BL/6 mice. (a) Thymus weight index; (b) spleen weight index. All data were expressed as means ± SD (*n* = 10). ^∗^ *p* < 0.05, ^∗∗^ *p* < 0.01, versus blank group; ^#^ *p* < 0.05, versus model group; ^$^ *p* < 0.05, ^$$^ *p* < 0.01, versus DDP group.

As shown in Figures 7(a) and 7(b), the thymus index and spleen index of the model group were much lower than those of the blank group (*p* < 0.05). This decrease of thymus index and spleen index of tumor-bearing mice was enhanced significantly by SB treatment in a dose-dependent manner, compared with the model mice (*p* < 0.05). Of note, DDP of 20 mg/k/d could inhibit the immunological function of mice through decreasing the indexes of spleen and thymus. However, these decreases were reversed remarkably by treatment with SB at the dose of 3.33, 6.67, and 10 g crude drug/kg/d. These results indicated that the flavonoids and scutebarbatines of SB might inhibit tumor growth which was related to the stimulation of the immune response.

### 3.4. SB Regulated Immunoregulatory Factor Levels of Serum in Lewis-Bearing C57BL/6 Mice

We used ELISA to determine the potential immunomodulatory activities of the flavonoids and scutebarbatines in SB by assessing the cytokine-production profiles. As can be seen in Table 2, there is a severe increase of IL-10, IL-17, ROR*γ*t, FOXP3, TGF-*β*1, and IL-6 in the serum of model mice compared to normal blank mice (*p* < 0.05 or *p* < 0.01). And there is a significant decrease of IL-2 and IFN-*γ*, compared to normal blank mice (*p* < 0.05 or *p* < 0.01). The high dosage of flavonoids and scutebarbatines (10 g/kg/d) could significantly reverse the levels of these cytokines to normal levels. Our findings indicated that flavonoids and scutebarbatines could regulate immunomodulatory molecules in Lewis tumor-bearing mice due to their immunomodulatory function.

### 3.5. SB Regulated the Protein Expressions of Immune Factors in Lewis-Bearing C57BL/6 Mice

**Figure 8:**
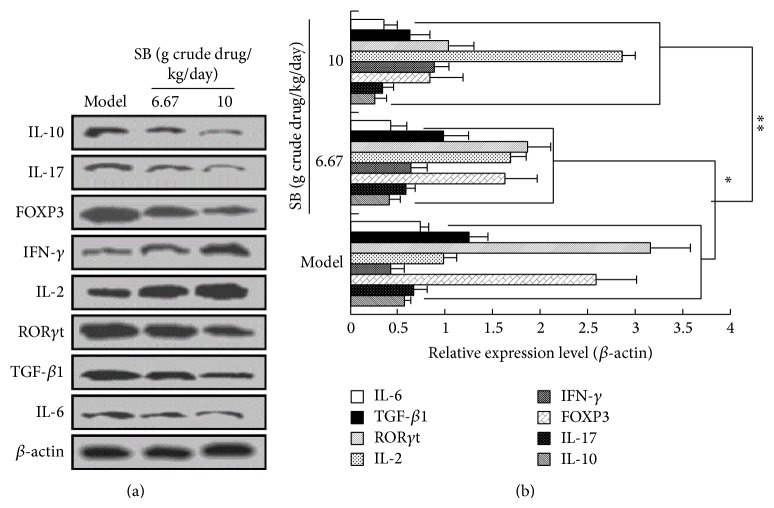
Regulation of SB on protein expressions of immune factors in Lewis-bearing C57BL/6 mice. (a) Western blot analysis; (b) relative protein expression levels. All data were expressed as means ± SD (*n* = 10). ^∗^ *p* < 0.05, ^∗∗^ *p* < 0.01, versus model group.

To further verify the ELISA results, western blot analysis was also performed to determine the protein expression of immune-related factors. As shown in Figure 8, we observed that SB treatment at the dose of 3.33, 6.67, and 10 g crude drug/kg/d could decrease significantly the protein expressions of IL-10, FOXP3, TGF-*β*1, IL-17, ROR*γ*t, and IL-6 while increasing remarkably protein expressions of IL-2 and IFN-*γ*, compared with the model mice (*p* < 0.05 or *p* < 0.01). These results supported the above conclusion that SB had a significant immunomodulatory effect contributing to its inhibition on tumor growth in Lewis tumor-bearing C57BL/6 mice.

## 4. Discussion

Medicinal herb SB has been reported to hold protective effects against rat liver tumorigenesis [17]. Compounds, flavonoids and scutebarbatines, were rich in the extract of SB and have been shown to contribute to the antitumor activity of SB [6]. In this study, we found that the flavonoids and scutebarbatines of SB could inhibit the tumor growth of Lewis tumor-bearing C57BL/6 mice and also held a significant immunomodulatory activity* in vivo*. These findings provide evidence for the application of SB in the treatment of tumor, especially in lung cancer.

It is reported that immunostimulation has been considered as one of potential mechanisms contributing to the inhibition of tumor growth. Traditional chemotherapy is beneficial against tumor growth, but its side effects such as immunosuppressive effects and toxicity limit its use in clinic [18]. The decrease of the relative spleen and thymus weight has been regarded as the most sensitive indicator of immunosuppression. Spleen index and thymus index, two immune parameters, are related closely to the immune function. In tumor-bearing mice, these two indexes were used usually for the evaluation of immune function [19].

Regulatory T cell (Treg) enrichment in the tumor microenvironment is regarded as an important mechanism of tumor immune escape [20]. In the tumor microenvironment, Tregs cells could inhibit the proliferation of tumor cells to exert an antitumor effect through suppressing CD4 + T cells and CD8 + T cell activation. The increase of Treg cells downregulated the number of Th17 cells and the levels of Th17-related cytokine IL-17 and retinoic acid receptor-related orphan receptor *γ*t (ROR*γ*t). FOXP3 was one of transcriptional repressors of transcription factor forkhead or winged helix family and was expressed in all the CD4 + Tregs. FOXP3 can regulate the differentiation and development of Treg cells. In Foxp3 gene knockout mice, the level of Treg cells was decreased significantly and then led to generate a large amount of chronic inflammatory cytokines. However, in our experiment, we observed that FOXP3 was increased in model mice. SB treatment could decrease this reduction. T helper cell 17 (Th17) is a new discovered subtype of effector T cells which is able to secrete IL-17. ROR*γ*t has been confirmed to be a specific transcription factor for Th17 cell differentiation [21–25].

Immunosuppressive molecules transforming growth factor-*β*1 (TGF-*β*1) and IL-10 favored generation of Treg and Th2-oriented responses that rendered CD8 (+) T cells dysfunctional [26]. Th2-related cytokines IL-10 and TGF-*β*1 were involved in tumour immune tolerance [27]. The levels of IL-10 and TGF-*β*1 in model mice were increased while the treatment of SB could decrease them. For cytokines IL-2 and IFN-*γ*, they were both related to Th1. We observed that SB could increase significantly their IL-2 level and IFN-*γ* level. These results suggest that SB exerts the immunomodulatory effects for the proliferation of tumor cells. It might be associated with regulating Th1/Th2 imbalance and Th17/Treg imbalance.

## 5. Conclusion

In conclusion, this study demonstrated for the first time the inhibition of SB including flavonoids and scutebarbatines on tumor growth* in vivo* and this function might be associated with immunomodulatory activity through regulating Th1, Th2, and Th17 activation. Our study provided direct experimental evidence to demonstrate that SB might act as a promising intervention agent for the application in the treatment of lung cancer.

## Figures and Tables

**Table 1:** The retention time, UVmax, and MS ion fragment information of flavonoids and scutebarbatines using HPLC-DAD and LC-ESI/MS/MS.

Number	Classification	Compound identity	*t*_*R*_ (min)	(−)ESI-MS *m*/*z*	UVmax (nm)	Ion fragments
1	Flavonoids	Scutellarin	29.14	461.32	225, 282, 334	433, 285, 174
2		Naringin	54.26	579.34	283	547, 519, 444, 332, 273, 171
3		Scutellarein	57.93	269.15	270, 320	268, 225, 148
4		Luteolin	64.15	285.13	210, 254, 349	257, 241, 175
5		Apigenin	65.77	269.02	210, 265, 334	267, 239, 223
6		Wogonin	72.81	285.07	200, 275	267, 239, 223

7	Scutebarbatines	Scutebarbatine A	80.53	557.60	220, 264	503, 473, 353, 257
8		Scutebarbatine B	92.20	556.17	220, 264	508, 478, 449, 356, 192

**Table 2:** Effect of flavonoids and scutebarbatines in SB on the levels of immune factors in Lewis tumor-bearing mice. All data were expressed as means ± SD (*n* = 9).

Group	Dosage (g crude drug/kg/day)	IL-10	IL-17	FOXP3	IFN-*γ*	TGF-*β*1	ROR*γ*t	IL-2	IL-6
		(pg/mL)	(pg/mL)	(pg/mL)	(ng/mL)	(ng/mL)	(pg/mL)	(ng/mL)	(pg/mL)
Blank	—	78.07 ± 3.32	7.48 ± 1.10	128.00 ± 6.83	39.90 ± 3.42	46.46 ± 2.87	7.07 ± 1.10	21.26 ± 3.74	8.00 ± 1.03
Model	—	137.14 ± 5.28^#^	15.79 ± 3.59^#^	164.70 ± 7.98^#^	20.66 ± 3.01^#^	75.20 ± 2.46^#^	20.62 ± 1.06^#^	12.35 ± 2.85^#^	11.82 ± 1.25^#^
DDP	20 mg/kg/d	84.33 ± 3.03^∗^	9.33 ± 2.12^∗^	127.10 ± 6.32	24.93 ± 4.28	63.77 ± 3.18	15.91 ± 3.12	11.94 ± 4.72	10.46 ± 1.16
	10	67.51 ± 3.11^∗^	9.83 ± 3.01^∗^	134.80 ± 8.03^∗^	45.70 ± 2.87^**∗∗**^	37.61 ± 2.32^∗^	8.02 ± 2.14^**∗**^	39.70 ± 4.04^**∗**^	9.61 ± 1.17^∗^
SB	6.67	82.73 ± 3.03	13.73 ± 4.16	149.10 ± 7.91	36.36 ± 4.27^**∗**^	53.33 ± 3.47	12.83 ± 3.58^**∗**^	28.85 ± 3.18	9.85 ± 1.11^∗^
	3.33	99.44 ± 3.12	14.93 ± 5.10	158.20 ± 8.35	32.40 ± 3.86	65.23 ± 3.24	15.79 ± 2.39	21.12 ± 3.19	10.97 ± 1.39

^∗^ *p* < 0.05, ^∗∗^ *p* < 0.01 versus model group; ^#^ *p* < 0.05 versus blank group.
